# Children of Parents With a Mental Illness—Stigma Questionnaire: Development and Piloting

**DOI:** 10.3389/fpsyt.2022.800037

**Published:** 2022-04-08

**Authors:** Lisa-Marie Dobener, Markus Stracke, Kathrin Viehl, Hanna Christiansen

**Affiliations:** Department of Clinical Child and Adolescent Psychology, Philipps University Marburg, Marburg, Germany

**Keywords:** children of parents with a mental illness, stigma by association, family stigma, COPMI-SQ, questionnaire, instrument development, piloting

## Abstract

Children of parents with a mental illness are a particularly vulnerable group as they have a high risk to develop a mental disorder themselves and those are associated with high stigma. Moreover, just like primary recipients of stigma, they are affected by the social taboo surrounding mental illness: they do not receive enough information, are often left alone with their problems, and are thus considered “invisible children”. In previous research, family stigma has only been assessed through general questionnaires for all family members. What has not yet been adequately investigated is how stigma difficulties affect the children of parents with mental illness in particular. To address these limitations, we developed the Children of Parents with Mental Illness-Stigma-Questionnaire (COPMI-SQ), a self-report instrument for young people aged 12–19 years, designed to assess young people's stigma experiences in daily life. Based on a systematic review preceding the questionnaire, we identified relevant stigma dimensions for children of parents with a mental illness that resulted in 93 items that according to theory were assumed to load on four different scales: experienced stigma, anticipated stigma, self-stigma, and structural discrimination. An expert discussion, and a comprehensibility analysis with the target group followed. In this paper, we report on the development process and initial pilot data (*N* = 32) on the psychometric properties of the COPMI-SQ. Item analyses via an item difficulty index, discriminatory power, as well as internal consistency analysis resulted in a revised instrument reduced to 67 items. We observed very high internal consistencies (between α = 0.868 and α = 0.975) for the subscales. The approach taken to develop the COPMI-SQ followed scientifically accepted principles by ensuring different construction phases and is considered a solid basis for further reliability and validity studies. The study is ongoing and undergoing a further validation investigation; dimensionality and factor structure will also be examined.

## Introduction

Approximately 17–25% of all children worldwide live with at least one parent who has a mental illness (1–4). Evidence shows that children of parents with a mental illness (COPMI) are exposed to serious and diverse risks compared to their peers of similar age (3). The high prevalence and known risks contrast with the very low visibility of these children (5). Mental illnesses of parents can influence the living environment of children and adolescents in many ways, and they carry various risks associated with reduced mental health, poorer academic achievement, and impaired social well-being (3, 6). Moreover, COPMI carry a significantly increased risk of developing mental health problems themselves (1). In addition to genetic factors and familial influences resulting from or accompanying the parental illness, there are environmental factors that contribute to whether children develop a mental illness themselves during their lives (1, 7). One social environment mechanism that influences the entire family system and is known to be relevant in terms of children's personal development, wellbeing, and help-seeking behavior is the stigma associated with a parent's mental illness (8, 9). Despite high prevalence rates of children of parents with a mental illness, they are remaining “invisible” due to the lack of recognition and formal identification within (adult) health systems (10, 11). Through the fear of stigma and negative repercussions of those children and their families, many of these children remain hidden (8, 9).

Although the appearance of stigma is liable to historical, cultural, and temporal changes, there is hardly a country, society, or culture in the world where mental illness is not stigmatized (12, 13). Stigma is an attribute leading to widespread social disapproval, and encompasses the negative effects of a label placed on any group (14, 15). It occurs in social situations, meaning that stigma does not reside in the person itself, but is the result of the social context and the perception of the public (16). People with a mental illness are frequently viewed as dangerous, unpredictable, incompetent, abnormal, and of weak character (17). While this is widely true for all types of mental disorders, the perception of different disorders varies and therefore the stigma attached to it: for instance, schizophrenia is associated with much more “dangerousness” than depression, resulting in greater social distancing from the public to the people it is associated with (18).

With regard to the stigma of mental illness, as with other stigmatized conditions, there is evidence that those affected themselves by mental illness are not the only individuals who suffer social stigma (19). Goffman, who raised the concept of stigma, has already outlined the phenomenon of the so-called *courtesy stigma* in 1963 (15). Nowadays, it is widely known as *stigma by association (SBA)* or *family stigma*, describing family members as also being exposed to stigma resulting from a family member's mental illness (20). An abnormality attributed to individual family members or the family as a whole is considered key to the development of family stigma (21). Another crucial factor in determining whether a SBA occurs appears to be the entitavity, i.e. the degree to which two or more people form a significant social unit (22, 23). The higher the entitavity, the greater the likelihood of being stigmatized on the basis of association (23). Intimate groups such as families are attributed the highest degree of entitavity (23)—the association with a stigmatized family member and one's own experience of stigmatization is therefore very likely.

Children and adolescents who grow up with parents with a mental illness are thus—due to their dependent and close relationship to the affected person—a particularly vulnerable group for stigma. They may face the stigma due to the parental mental illness itself, and due to associated peculiarities and “otherness” within their families as described above (21, 24, 25). Negative effects in the affected children and young people arise both from the actual stigma experienced as well as the fear of being stigmatized and the internalization of stigmatizing attitudes toward them and their families (24, 26, 27).

There are many theories clarifying the various facets of stigma for people with a mental illness. Yet there is no comprehensive theoretical model for SBA. Research suggests that family stigma is no monolithic phenomenon, because it varies depending on the relationship the family member has with the person with the mental illness: studies have shown that parents of children with a mental illness are more likely to experience the stigma of neglect and blame for their children's disease onset (12, 28), and the siblings of children with a mental illness and spouses of someone with a mental illness are more likely to experience the stigma of blame for the persistence of their relative's disease (12); COPMI are more likely to face the stigma of “contamination”—meaning that the general population believes that parental illness, and especially regarding COPMI, is passed on to children (29). However, studies in this regard are very sparse. By describing a “contamination” stigma, authors are often referring to the public perception of stigma toward COPMI [e.g., (30, 31)], and are thereby missing out to understand children's lived experiences. Thus, these studies possess little informative value if we hope to understand how children perceive and experience family stigma. A recent integrative review on the evidence of stigma concepts for children of parents with a mental illness has shed light on those limitations by identifying stigma-related experiences and outcomes as reported by parents and children (29). Nevertheless, this review shows that a concept which includes the various dimensions of stigma experiences of children whose parent has a mental illness is missing. The results of the review show that children report feelings of embarrassment, shame, and the need to hide their parental mental illness, but the authors do not integrate those findings into an overall framework of different stigma dimensions.

To fill this gap, we have conducted a systematic review to analyze the COPMI's experiences of stigma and to identify specific stigma dimensions and their characteristics for this specific target group (28). Our review resulted in four stigma dimensions: (1) *Experienced SBA* describes personally experienced prejudice and discrimination (32, 33); (2) *anticipated SBA* incorporates expectations that others will devalue and discriminate against them in the future (32); (3) *affiliate stigma* describes the self-stigma of people associated with a mental illness, i.e. the internalization of public stigma (34); and (4) *structural discrimination*, entailing social institutions and ideological systems that reproduce and maintain the stigmatized status (14).

In a literature search, we identified nine scales measuring mental illness SBA or family stigma (34–42) as well as one family-experiences interview with a stigma subscale (43). Most of the scales only measure the stigma component self-stigma/internalized stigma (34–36, 40). One of the scales was a slightly modified version of a scale constructed for primary stigma recipients, i.e., people with a mental illness (39). Another scale was developed to measure the SBA of relatives of patients with schizophrenia (42); the others were not restricted to a specific relative's mental illness. Six scales as well as the interview schedule stigma scale were designed for all family members, while two of the scales measure the stigma parents of children with a mental illness suffer from (40, 41). None of the scales was constructed or validated for minor children. The items' wording is often awkward [e.g., “*Most people believe their friends would not visit them as often if a member of their family were hospitalized for serious mental illness”* (37)]. In addition, the way the items are phrased often presuppose a great deal of basic knowledge of certain emotional and cognitive processes, such as “*I feel emotionally disturbed because I have a family member with mental illness/intellectual disability”* (34), or do not seem stigma-specific: “*Having a family member with mental illness/intellectual disability imposes a negative impact on me”* (34).

Furthermore, we know that stigma varies depending on the individual's role within the family, and none of the established instruments combines all the stigma dimensions highlighted in the review that are relevant to children with a mentally ill parent. Our systematic review has revealed aspects of stigma that are quite specific to their role as children, for example, being bullied and teased in school, being isolated because of feeling ashamed and therefore not inviting friends home, being afraid of passing on the illness to another generation, being responsible as a child for the parent's wellbeing when psychiatrists turn them away, having nobody to talk to, and not getting enough information about their parent's mental illness.

Overall, for children of parents with a mental illness, no suitable instrument exists yet to measure their stigma experiences, expectations, self-stigma and the stigma's structural dimensions—especially none considering the special role of children. The current study aimed to describe the development and initial pilot data on our newly developed Children of Parents with a Mental Illness—Stigma Questionnaire (COPMI-SQ). This scale can provide important information to better understand the complex phenomenon of stigma by association among young people and shed light on experiences and needs of this group in intervention studies or anti-stigma campaigns.

## METHODS

**Figure 1:**
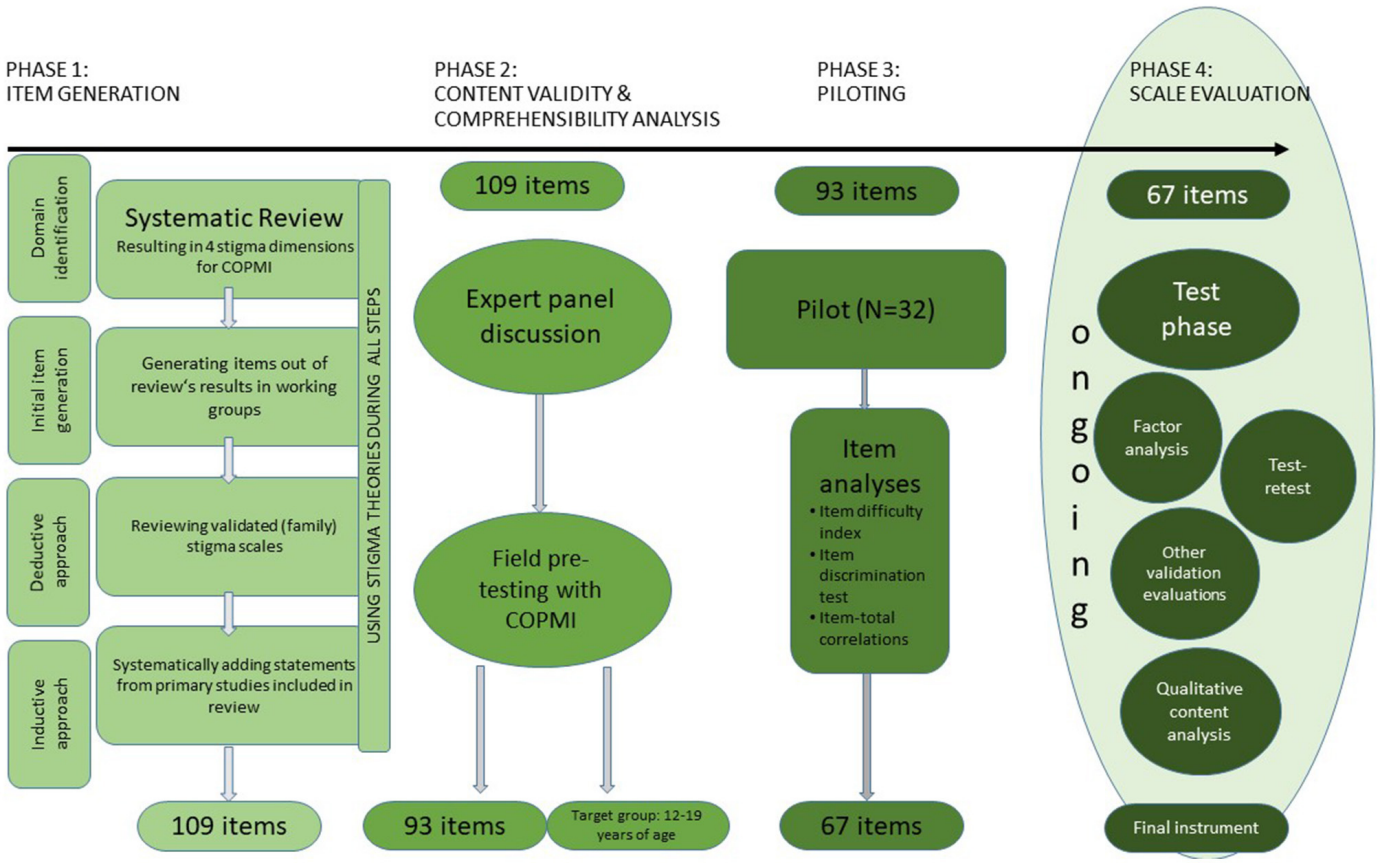
Phases of development of the instrument measuring stigma in COPMI (COPMI-SQ).

Figure 1 shows the different stages of development for the COPMI-SQ. We have oriented ourselves to two guidelines for the development of new scales (44, 45). We are currently reporting on phase 3 and present our pilot data. Phase 4 is still under way.

### Ethics

Research with children and adolescents is always in a field of tension between the respect of the various rights of children and their special need for protection. In recent decades, a paradigm shift has occurred within research, in which children are now rather seen as “*moral agents in their own right”* (46). As a result, more research is now being conducted with children to increase their participation. Researchers and adults in general cannot assume that their view of the world is congruent with that of children (46). In order to comply with the UN Convention on the Rights of the Child, we consider it of fundamental importance to interview children and adolescents themselves when it comes to their life experiences and the intention to improve their living conditions. Since it can be assumed that stigma may affect a lot of children of parents with mental illness in particular, it is necessary to obtain their perspectives before interventions are directed past their needs.

In order to ensure the special protection of children under guardianship, we followed the guidelines of the Central Ethics Committee of the German Medical Association (47), which stipulates a need for an informed consent both from their parents with a mental disorder and the children themselves in age-appropriate language prior to study participation. We considered the costs and benefits of a survey, which could be potentially burdensome and concluded that the results of the survey offered great insights into the experiences of this population which could be beneficially used for further interventions for this population. However, since it can be assumed that children of parents with mental illness are particularly exposed to stresses that may also be related to stigmatizing experiences, they were offered to contact the persons responsible for the study, who are child and adolescent psychotherapists, at any time. In addition, they were advised to contact any trusted adult person in case of need. Furthermore, prior to study participation, they were informed that they could withdraw from their participation in the online study at any time and without giving reasons, up to when the data were analyzed.

The study was approved by the Ethics Committee of the Psychology Department of Philipps University of Marburg (study approval number: 2020-20).

### Inclusion Criteria

Young people were eligible to participate in the study as long as they were between 12 and 19 years old and one or both parents had a mental illness. Information regarding the illness was obtained through a self-report by the parents. Otherwise, there were no exclusion criteria.

### Phase 1: Item Generation

The first step toward designing this new instrument, the systematic review, resulted in four different dimensions of family stigma in COPMI, i.e. experienced SBA, anticipated SBA, affiliate stigma (self-stigma), and structural discrimination. Those findings were presented by a PhD student to 50 scientific collaborators in Clinical Psychology and Clinical Child and Adolescent Psychology working groups of Marburg University, Department of Psychology, including pre-, post-docs, and professors. Post-docs and professors further possessed licenses as psychotherapists and PhD students were psychotherapists in training. Quotes from primary studies based on a systematic review with the instruction to collect ideas for items for a survey were presented to all participants. Initial items that may be used in a survey for this population and for the different dimensions were generated through group discussions.

The inductively generated items were then compared with already validated instruments measuring (family) stigma (see above) to apply formulations already established whenever possible. Using the established stigma scales, the generated items were developed further and supplemented in their formulation.

To ensure that every aspect of stigma was covered, the first pool of items was then supplemented by adding further inductively formed items for every (sub-)sub-category identified in the review [for further information see Dobener et al. (28)]. Statements from the included primary studies were included also to reflect as accurately as possible the comments of the target group members themselves in the items. For each category identified in the review, 5–15 items were formed. A total of 109 items was generated in phase 1.

### Phase 2: Content Validity and Comprehensibility Analysis

#### Expert Discussion

The entire item pool was then reviewed and discussed by a panel of experts from our and other known working groups, with knowledge in developing questionnaires as well as in the field of stigma. The experts were asked to rate all items according to the extent to which they seemed to be an appropriate measure of the intended construct. They were also asked to provide open-ended feedback. Items agreed upon by at least two of the three experts were retained or reworded according to their comments. This ensured that only items that were understandable and meaningful in terms of both content and language were used. Through this process, the item pool was reduced to 96 items since the expert panel revealed problems like overly complex wording, repetition, or other violations of “best-practices” regarding item structure (48).

#### Comprehensibility Analysis With Children

In a further step, five children and adolescents of different ages whose parents have a mental illness filled in the questionnaire to check comprehensibility. They completed the COPMI-SQ in the presence of the author L-MD, to enable immediate feedback on any difficulties in understanding. In addition to the comprehensibility check, this allowed us to see whether filling in the COPMI-SQ was associated with any mental stress when confronted with the effects of their parents' illness. According to the children's feedback, some items were reworded, and three were removed entirely.

At the end of this second phase, the item pool was reduced to 93 items, of which 91 were closed questions and two were open-ended. Due to the complexity of wording and the general need for any mental illness concept as well as children's feedback, we found our survey to be most suitable for children and adolescents aged 12–19 years. For younger children, the language and delivery as an online survey does not seem the right format.

#### Phase 3: Piloting

Our questionnaire was piloted in an online survey with a sample of children and adolescents having at least one parent with a mental illness.

The study was conducted using the online platform SosciSurvey (49) between March and May 2021. All participants were part of a convenience sample—either contacted as part of an ongoing study [COMPARE-family, see (4, 50), via university mailing lists or clinical institutions; e.g., psychiatric clinics, psychotherapy outpatient clinics]. Participants could participate in a raffle of several gifts as study reimbursement. Inclusion criteria for the COPMI-SQ-validation were having a parent with a mental illness and being 12–19 years of age. Participants were not excluded if they themselves had a mental illness.

#### Measures

Participants responded to questions pertaining to socio-demographic characteristics (age, school grade, living situation, etc.) and questions about their parent's mental illness and their own health status.

##### COPMI-SQ

Stigma experiences in COPMI were measured via the 93 items in the newly developed COPMI-SQ. Participants were first asked to complete two open-ended sentences: “*I think about people with a mental illness …”* and “*Others think about people with a mental illness …”*. Afterwards, they were asked to rate each of the 91 closed items on a visual analog scale (VAS). The VAS consisted of a line with verbal anchors (“strongly disagree” on the far left and “strongly agree” on the far right). All of the items were scored from 1 to 101, with 1 meaning no agreement at all, 101 meaning full agreement. Most of the items were preceded by an item root like “*Because my mother/my father has a mental illness,…”*, others were presented separately. The questionnaire consists of 26 reversed coded items, whose agreement represented a low stigma experience to control for and identify acquiescence response bias (51), see Supplementary Table S1. For all other items, the higher the agreement, the greater the experience of stigma.

The first theoretically assumed dimension, *experienced stigma by association (SBA)*, consisted of 18 items representing varied aspects of directly experienced stigmatization as a result of the parental mental illness; for example, experiencing rejection by others, inappropriate and hurtful language or warnings, and obviously discriminatory behavior such as bullying.

The second theoretically assumed dimension, *anticipated SBA*, was captured across 20 items and included fears of stigmatizing behavior or the attitudes of others if they found out about the parental mental illness.

For the third theoretically assumed dimension, *self-stigma*, 23 items were constructed to capture different facets of self-stigma: the feeling of being “contaminated” by the parental illness, the shame associated with the parental illness, feelings of guilt, and feeling generally inferior compared to other people.

The fourth and last theoretically assumed dimension represents the experienced *structural discrimination* and was captured by 30 items. This included discrimination in the education system, the health system, the media, and discrimination in society as a whole. Our instrument's entire structure and all its items are found in Supplementary Table S1.

#### Statistics

##### Sample Size

We aimed to recruit about 200 participants to validate the scale's psychometric properties. The sample size of 200 is considered adequate for validating an instrument, i.e., for item analyses, and factor analyses to examine the questionnaire's multidimensionality (52, 53).

##### Missing Values and Outliers

The online survey was designed so that all items had to be answered. Thus, none of the included cases had any missing values. However, there was a filter question addressing parental hospitalization. If this was answered positively, all the subsequent questions were posed only to COPMI whose parent(s) had been hospitalized; this group formed a smaller subsample of only 18 children. All items were analyzed for univariate outliers using boxplots. Conspicuous cases were assessed as realistic after examination and were in a generally inconspicuous range across the mean values achieved; thus they were not excluded from further analyses.

#### Item Analyses

##### General Procedure

The item analyses were carried out in three phases. In the first phase, items were checked at the subscale level for appropriate item difficulty and discriminatory power, and subscales were adjusted by removing inappropriate items. Due to content considerations, some psychometrically inappropriate items were retained. In the second phase, the adjusted subscales were combined into an overall scale, the items were rechecked for their characteristic values, and unsuitable items were removed accordingly to improve global consistency and increase the suitability of the overall instrument. In the third phase, the subscales of the COPMI-SQ were adapted to the structure of the final overall instrument, and the final instrument's internal consistency was calculated again. Statistical analyses were carried out using SPSS, version 20.

##### Item Difficulty Index

Given that the COPMI-SQ is intended to allow differentiation across a broad spectrum of trait values, we aimed for a distribution of item difficulties of 20 < *P*_*i*_ <80 [see (54)]. Since extremely difficult, or very easy items hardly allow differentiation between test persons, items with values below 20 and above 80 were considered more closely. Items of very low or extreme difficulty were checked for their discriminatory power and their retention or removal from the item pool was supplemented with content considerations. The exact reasons for retaining or deleting a given conspicuous item are provided in Supplementary Table S1.

##### Discriminatory Power

The item discrimination index was calculated using the corrected item-total-correlation. Values of *r*_*it*_ between 0.3 and 0.5 were considered acceptable; 0.5 < *r*_*it*_ <0.7 is considered good discriminatory power (54). All items whose discriminatory power was below 0.3 were scrutinized, and usually removed to ensure high internal consistency of the subscales and the overall instrument. However, those items with cut-off values below 0.3 were checked for their relevance to the content and either deleted or reformulated when evaluating strongly relevant content. They were then removed one after the other and new item-total correlations were calculated in each case to see how the items interacted. The order of removal of the items and content-related considerations is illustrated in Supplementary Table S1.

##### Internal Reliability

Cronbach's alpha was calculated to measure the COPMI-SQ's internal reliability (55). This should attain a value of at least 0.7 for the subscales—the widely accepted criterion for Cronbach's alpha [e.g., (56)].

## Results

### Sample

All participants (*N* = 32) were between 13 and 19 years of age (M = 15.75, SD = 2.05). Sixty-five percent of the participants were female. The COPMI usually lived together with their parent with a mental illness (93.8%). Three COPMIs reported having both parents with a mental illness. Mothers were most often affected (60%). The most common parental diagnosis was a mood disorder such as bipolar disorder and depression (maternal: 54.2%; paternal: 82.3%), followed by anxiety disorders (maternal: 16.7%; 11.8% paternal). Almost half of the children stated to have already suffered from a mental illness themselves (46.9%). These were, similar to the parental diagnoses, usually mood disorders (32%). All the sample characteristics are provided in Table 1.

**Table 1:** Familial and COPMI-specific sample characteristics T1, *N* = 32.

**Characteristics**	**Categories**	***n* (%)**
Gender^a^	Female	21 (65.6)
	Male	10 (31.3)
	Diverse	1 (3.1)
Age^a^	M (years)	15.75
	SD	2.05
	Min	13
	Max	19
Living situation^b^	Together with ill parent	30 (93.8)
	Without ill parent	2 (6.2)
Parental mental illness^b, c^	Mother	21 (58.3)
	Father	14 (38.9)
Diagnoses mother^b, c^	Mood disorders	13 (54.2)
	Phobia/anxiety disorder	4 (16.7)
	Personality disorders	3 (12.5)
	Posttraumatic stress disorder	1 (4.1)
	Burn-out	1 (4.1)
	Obsessive compulsive disorder	1 (4.1)
	Schizophrenia	1 (4.1)
Diagnoses father^b, c^	Mood disorders	14 (82.3)
	Phobia/anxiety disorder	2 (11.8)
	Pain disorder	1 (5.9)
Children's mental illness^a^	Yes	15 (46.9)
	No	17 (53.1)
Diagnoses child^a, c^	Mood disorder	8 (32.0)
	Phobia/anxiety disorder	4 (16.0)
	AD(H)D	3 (12.0)
	Eating disorder	2 (8.0)
	PTSD	1 (4.0)
	Autism spectrum disorder	1 (4.0)
	OCD	1 (4.0)
	Sleeping disorder	1 (4.0)
	Attachment disorder	1 (4.0)
	Not to be classified	3 (12.0)

*n = stated characteristics*.

a *Information is based solely on information provided by COPMI themselves*;

b *Information is based solely on information provided by parents*;

c *Multiple answers possible*.

As COPMI are an exceedingly difficult target group to reach (57, 58), we were unable to recruit enough COPMI to assess decisively the questionnaire's psychometric goodness of fit, despite intensive recruitment efforts. Nevertheless, according to Johanson and Brooks (59), 30 representative participants from the population of interest suffice for a pilot study to test a new instrument in terms of preliminary item analyses, estimates of internal consistency and proportions of people responding to specific options. For factor and further analyses, more participants are needed, so we focused on the item analyses, especially to reduce the number of items requiring further validation and evaluation of the COPMI-SQ, in order to make the questionnaire more practical and reduce participation thresholds as well as raise the quality of the instrument.

### Item Analysis on Subscale Level

#### Experienced SBA Subscale

Initial Cronbach's alpha equalled 0.940 (see **Table 4**). In the experienced SBA scale, two items revealed low item difficulty, and one item a low item-total correlation. The item with the low item-total correlation was thus removed. However, the two items of low item difficulty were retained after content considerations. Both had a discriminatory power above 0.7. The item “*Because my mother/my father has a mental illness, my friends no longer want to be friends with me”* was retained because this aspect addressing the loss of friendship stood out in the review as a relevant experience for COPMI, and that aspect was not covered by any other item. Similar considerations applied to the item “*Because my mother/my father has a mental illness, I'm bullied at school/university/work”*: the aspect of bullying has been described in the literature, and as it is not covered by any other item in the questionnaire, we retained this item.

#### Anticipated SBA Subscale

In the anticipated SBA subscale (initial Cronbach's alpha of 0.927), four items revealed a low item-total-correlation; one of them also revealed low difficulty. The item with low discriminatory power and low difficulty was removed first. After considering the content, the other items with too little discriminatory power were also removed one after the other, because they were either covered by other items or classified as non-essential. The step-by-step removal of the items led to an increased item-total correlation within the subscale.

#### Affiliate Stigma Subscale

In the Affiliate Stigma subscale with an initial alpha of 0.904, two items were conspicuous due to low difficulty and two items due to low discriminatory power. The item with negative discriminatory power was removed first, as it seems to be misleading. Furthermore, its content was already covered by other items. The other item with low discriminatory power was also removed. The item “*Because my mother/father has a mental illness, I don't think I should have children of my own later”* was retained despite its low difficulty because it measures the belief in contamination, which has consequences for future plans, and is not covered by any other items. Furthermore, the other item of low difficulty “*I feel like I'm carrying around a sign: He/she has a mother/father with a mental illness”* was also retained because, it was adapted from an evaluated stigma scale and, additionally, the aspect of “contamination” is otherwise not sufficiently embodied in comparison to “inferiority.”

#### Structural Discrimination Subscale

Out of the Structural Discrimination subscale with an initial alpha of 0.817, 16 items revealed low discriminatory power. Of these, one had very high and one very low item difficulty. The first ones deleted stepwise were those three items with negative item-total correlation, indicating that they measured something completely different. Further items of very low discriminatory power were then successively removed, starting with those intended to measure discrimination within the mental health system. This was because it was clearly overrepresented compared to the other dimensions of the subscale.

The item “*At school, I'd like to learn more about mental illness”* was retained despite its low discriminatory power, firstly because it was on the edge of acceptance (r_it_ = 0.295), secondly, in order to maintain the proportions to the other structural stigma dimensions to some extent, and thirdly, because the content of the item was attributed a special significance concerning possible interventions. For the media as a source of structural discrimination, the item “*In the media, mental illness is portrayed negatively, which contributes to many people developing prejudices”* was retained despite its low item-total-correlation; for the reason of having at least two items representing this source of discrimination. In addition, we reworded the item in order to avoid multidimensionality as follows “*Mental illness is portrayed negatively in the media.”*

### Item Analysis on Total Scale

In the second phase of the item analyses, i.e., when assessing the internal reliability of the overall instrument, one item in the Affiliate Stigma scale and an item in the Structural Discrimination scale showed low item-total correlations and were therefore deleted. Supplementary Table S1 shows which items were affected.

### Revised COPMI-SQ

Our instrument was reduced to a total of 67 items after the three phases of item analyses. The revised instrument is illustrated in Table 2. The final version of the *Experienced SBA* subscale now consists of 17 items, with Cronbach's alpha = 0.948 and item-total-correlations from good (lowest 0.395 for the item “*There are people I can talk to about my fears and worries.”*) to very high (highest 0.905 for item “*Because my mother/father has a mental illness, my classmates/colleagues/work colleagues tease me.”*). The final subscale *Anticipated SBA* (Cronbach's alpha = 0.949) shows item-total correlations between 0.426 (item “*If others found out about my mother's/father's illness, it wouldn't change their behavior toward me”)* and 0.877 (item “they'd bad-mouth me.”), see Supplementary Table S1. The final subscale of *Affiliate Stigma* consists of 19 items, with item-total correlations ranging between 0.449 (item: “I'm just a normal kid like any other.”) to 0.820 (item: “Because my mother/my father has a mental illness, I think there's something wrong with me”), and with Cronbach's alpha = 0.933. The item-total-correlations for the final subscale of *Structural Discrimination (STD)* are the lowest compared to the other subscales, and range from 0.282 (item “*In media, mental illness is appropriately portrayed”*), which is actually below acceptable discriminatory power, but will be kept to ensure the media source of discrimination is represented, to 0.705 (item: “*In school, I don't feel disadvantaged because of my mother's/father's illness”*). The revised STD subscale results in a Cronbach's alpha of 0.868.

**Table 2:** Overview of the revised COPMI-SQ after item analyses.

**New itemname**	**Original item**	**Translation**	**Intended theoretical dimension**	**Scoring**
**Experienced SBA**				
	Preceded by: **Weil meine Mutter/mein Vater eine psychische Erkrankung hat**, **…**	Preceded by: **Because my mother/my father has a mental illness**, **…**		
ESBA_01	machen sich andere über meine Mutter/meinen Vater lustig.	others make fun of my mother/father.	hostile behaviors of others	1–101
ESBA_02	reden andere hinter meinem Rücken über die Erkrankung meiner Mutter/meines Vaters.	others talk about my mother's/father's illness behind my back.	hostile behaviors of others	1–101
ESBA_03	lästern andere über mich.	others say awful things about me.	hostile behaviors of others	1–101
ESBA_04	lachen andere mich aus.	others laugh at me.	hostile behaviors of others	1–101
ESBA_05	tratschen andere das weiter.	others gossip about it.	hostile behaviors of others	1–101
ESBA_06	gehen andere mir aus dem Weg.	others avoid me.	withdrawal and rejection	1–101
ESBA_07	haben andere Angst vor meiner Mutter/meinem Vater oder mir.	others are afraid of my mother/father or me.	inappropriate language and contents	1–101
ESBA_08	möchten meine Freund*innen nicht mehr mit mir befreundet sein.	my friends no longer want to be friends with me.	withdrawal and rejection	1–101
ESBA_09	wollen sich meine Mitschüler*innen / Kommiliton*innen/ Arbeitskolleg*innen nicht mehr mit mir treffen.	my classmates/ colleagues/ work colleagues no longer want to get together with me.	withdrawal and rejection	1–101
ESBA_10	ärgern mich meine Mitschäler*innen/ Kommiliton*innen/ Arbeitskolleg*innen.	my classmates/colleagues/work colleagues aggravate me.	hostile behaviors of others	1–101
ESBA_11	werde ich in der Schule / Uni / auf der Arbeit gemobbt.	I'm bullied at school/university/work.	hostile behaviors of others	1–101
ESBA_12	wussten andere nicht, wie sie passend darauf reagieren / damit umgehen sollten.	others did not know how to react to or deal with it appropriately.	inappropriate language and contents	1–101
ESBA_13	haben andere mir geraten, selbst keine Kinder zu bekommen.	others have advised me not to have children myself.	inappropriate language and contents	1–101
ESBA_14	haben andere verletzende Sachen über mich oder meine Mutter/meinen Vater gesagt.	others have said hurtful things about me or my mother/father.	hostile behaviors of others	1–101
ESBA_15	Es gibt Leute, mit denen ich über meine Ängste und Sorgen reden kann.	There are people I can talk to about my fears and worries.	withdrawal and rejection	101–1
	**Separate items without an item root**			
ESBA_16	Andere Leute möchten nicht mit mir über die Erkrankung meiner Mutter/meines Vaters sprechen.	Other people don't want to talk to me about my mother's/father's illness.	withdrawal and rejection	1–101
ESBA_17	Wenn ich wegen der Erkrankung meiner Mutter/meines Vaters Hilfe brauche, gibt es Personen. mit denen ich sprechen kann.	If I need help because of my mother's/father's illness, there are people I can turn to.	withdrawal and rejection	101–1
**Anticipated SBA**				
	Preceded by: **Wenn andere von der Erkrankung meiner Mutter/meines Vaters erfahren würden,…**	Preceded by: **If others found out about my mother's/ father's illness,…**	
ASBA_01	würden sie sich über meine Mutter/meinen Vater lustig machen.	they'd make fun of my mother/father.	fearing hostile behaviors	1–101
ASBA_02	würden sie hinter meinem Rücken schlecht über die Erkrankung meiner Mutter/meines Vaters reden.	they'd talk badly about my mother's/father's illness behind my back	fearing hostile behaviors	1–101
ASBA_03	würden sie über mich lästern.	they would badmouth me.	fearing hostile behaviors	1–101
ASBA_04	würde das an ihrem Verhalten mir gegenüber nichts ändern	it wouldn't change their behavior toward me.	fearing hostile behaviors	1–101
ASBA_05	würden sie mich auslachen.	they'd laugh at me.	fearing hostile behaviors	1–101
ASBA_06	würden sie das für sich behalten.	they'd keep it to themselves.	fearing hostile behaviors	1–101
ASBA_07	würden sie mir aus dem Weg gehen.	they'd avoid me.	fearing lack of understanding rejection	1–101
ASBA_08	würden sie Angst vor meiner Mutter/meinem Vater oder mir bekommen	They'd become afraid of my mother/father or me.	fearing of negative attitudes and ascriptions	1–101
ASBA_09	würden sich meine Mitschüler*innen/ Kommiliton*innen/ Arbeitskolleg*innen nicht mit mir treffen wollen.	my classmates/ fellow students/ colleagues would no longer want to get together with me.	fearing lack of understanding rejection	1–101
ASBA_10	würden mich meine Mitschüler*innen/ Kommiliton*innen/ Arbeitskolleg*innen ärgern.	my classmates/ fellow students/ colleagues at work would get angry with me.	fearing hostile behaviors	1–101
ASBA_11	würde ich in der Schule / Uni / auf der Arbeit gemobbt werden.	I'd be bullied at school/university/work.	fearing hostile behaviors	1–101
ASBA_12	würden andere mir raten, selbst keine Kinder zu bekommen.	others would advise me not to have children myself.	fearing of negative attitudes and ascriptions	1–101
ASBA_13	würden sie verletzende Sachen über mich oder meine Mutter/ meinen Vater sagen.	they'd say hurtful things about me or my mother/father.	fearing hostile behaviors	1–101
	Preceded by: Wenn Fachleute (Jugendamt/Psycholog*innen/Sozialarbeiter*innen. etc.) von der Erkrankung meiner Mutter/meines Vaters erfahren würden. **…**	Preceded by: **If professionals (youth welfare office/psychologists/social workers. etc.) found out about my mother's/father's illness**. **…**		
ASBA_14	könnte ich weiterhin zu Hause wohnen bleiben.	I could still keep living at home.	fearing of negative attitudes and ascriptions	101–0
ASBA_15	bringe ich selten neue Freund*innen mit nach Hause, aus Angst. dass sie dann nicht mehr mit mir befreundet sein wollen.	I rarely bring new friends home for fear that they won't want to be friends with me anymore.	fearing lack of understanding and rejection	1–101
	**Seperate item without an item root**			
ASBA_16	Ich habe kein Problem damit, meinen Freund*innen meine (erkrankte) Mutter/meinen (erkrankten) Vater vorzustellen.	I have no problem introducing my (ill) mother/father to my friends.	fearing any harmful reaction	101–1
**Affiliate Stigma**				
	Preceded by: **Weil meine Mutter/mein Vater eine psychische Erkrankung hat**, **…**	Preceded by: **Because my mother / my father has a mental illness**, **…**		
AS_01	denke ich, mit mir stimmt etwas nicht.	I think there's something wrong with me.	beliefs of being inferior	1–101
AS_02	versuche ich, mich besonders normal und unauffällig zu verhalten, damit andere mir nicht anmerken, dass ich nicht normal bin.	I try to act particularly normal and inconspicuous so that others do not notice that I am not normal.	beliefs of being contaminated	1–101
AS_03	nehmen andere wahr, dass ich anders / komisch bin.	others notice that I am different/weird.	beliefs of being contaminated	1–101
AS_04	habe ich Angst, mich anstecken zu können.	I am afraid of catching the illness.	beliefs of being contaminated	1–101
AS_05	denke ich bei kleinsten Anzeichen, dass ich dieselbe Erkrankung habe wie meine…	I think at the slightest sign that I have the same illness as my mother/father.	beliefs of being contaminated	1–101
AS_06	denke ich, dass ich später keine eigenen Kinder bekommen sollte.	I don't think I should have children of my own later on.	beliefs of being contaminated	1–101
AS_07	fühle ich mich weniger wert.	I feel less worthy.	beliefs of being inferior	1–101
AS_08	ist meine Familie nicht richtig.	my family is not right.	beliefs of being inferior	1–101
AS_09	fühle ich mich schuldig.	I feel guilty.	beliefs of being inferior	1–101
AS_10	schäme ich mich.	I feel ashamed.	beliefs of being inferior	1–101
	**Seperate items without an item root**		
AS_11	Ich fühle mich, als würde ich ein Schild mit mir herumtragen: “Er/Sie hat eine Mutter/einen Vater mit einer psychischen Erkrankung”	I feel like I'm carrying around a sign: “He/she has a mother/father with a mental illness”.	beliefs of being contaminated	1–101
AS_12	Ich bin ein ganz normales Kind, wie jedes andere auch.	I'm just a normal kid like any other.	beliefs of being inferior	101–1
AS_13	Weil ich so ein schwieriges Kind bin, ist meine Mutter/ ist mein Vater erkrankt.	Because I am such a difficult child, my mother/ father has become ill.	beliefs of being inferior	1–101
AS_14	Ich bin (mit-)verantwortlich dafür, dass sich der Zustand meiner Mutter/meines Vaters nicht verbessert.	I am (co-)responsible for the fact that the condition of my mother/father isn't improving.	beliefs of being inferior	1–101
AS_15	Ich muss die Erkrankung meiner Mutter/meines Vaters geheim halten.	I have to keep my mother's/father's illness a secret.	beliefs of being inferior	1–101
AS_16	Wenn ich die Erkrankung meiner Mutter/meines Vaters beschreibe, spiele ich die Schwere der Erkrankung herunter.	When I describe my mother's/father's illness, I downplay the severity of it.	beliefs of being inferior	1–101
AS_17	Mir ist es peinlich, dass meine Mutter/mein Vater eine psychische Erkrankung hat.	I'm embarrassed that my mother/father has a mental illness.	beliefs of being inferior	1–101
AS_18	Ich schäme mich dafür, dass meine Mutter/mein Vater nicht wie andere Mütter/Väter ist.	I'm ashamed that my mother/father isn't like other mothers/fathers.	beliefs of being inferior	1–101
AS_19	Wenn meine Mutter/mein Vater wegen ihrer/seiner Erkrankung verurteilt wird, fühle ich mich auch verurteilt.	If my mother/father is judged because of her/his illness, I feel judged too.	beliefs of being contaminated	1–101
**Structural Discrimination**				
	Preceded by (after a filter question whether the parent ever has been hospitalized before): **Wenn meine Mutter/ mein Vater aufgrund der psychischen Erkrankung im Krankenhaus war**, **…**	Preceded by: **When my mother/father was in hospital because of the mental illness**, **…**		
STD_01	konnte ich das Personal immer ansprechen, wenn ich Fragen zur Erkrankung meiner Mutter/meines Vaters hatte.	I could always approach the staff if I had any questions about my mother's/father's illness.	health care system	101–1
STD_02	hätte ich gerne mehr Informationen vom Krankenhauspersonal bekommen.	I'd have liked to get more information from the hospital staff.	health care system	1–101
STD_03	fühlte ich mich vom Krankenhauspersonal gut einbezogen und informiert.	I felt well integrated and informed by the hospital staff.	health care system	101–1
STD_04	fühlte ich mich, als wäre ich dort unerwünscht.	I felt like I was unwanted there.	health care system	1–101
STD_05	war die Beziehung zwischen mir und dem Krankenhauspersonal gut.	the relationship between me and the hospital staff was good.	health care system	101–1
	**Seperate Item without an item root:**			
STD_06	Meiner Mutter/meinem Vater wurde durch das Gesundheitssystem nicht genug geholfen.	My mother/father wasn't helped enough by the health system.	health care system	1–101
	Preceded by: **In der Schule…**	Preceded by: **At school.…**		
STD_07	würde ich gerne mehr über psychische Erkrankungen erfahren.	I'd like to learn more about mental illness.	educational system	1–101
STD_08	kann ich mit meinen Lehrer*innen über die Erkrankung meiner Mutter/meines Vaters sprechen.	I can talk to my teachers about my mother's or father's illness.	educational system	1–101
STD_09	gehen die Lehrer*innen auf mich und meine Schwierigkeiten zu Hause ein.	the teachers respond to me and my difficulties at home.	educational system	101–1
STD_10	fühle ich mich wegen der Erkrankung meiner Mutter/meines Vaters nicht benachteiligt.	I don't feel disadvantaged because of my mother's/father's illness.	educational system	101–1
	Preceded by: **In den Medien…**	Preceded by: **In the media**,		
STD_11	werden psychische Erkrankungen angemessen dargestellt.	Mental illness is portrayed appropriately.	media	101–1
STD_12	werden psychische Erkrankungen negativ dargestellt.	Mental illness is portrayed negatively.	media	1–101
	**Seperate items without an item root**		
STD_13	Ich erhalte von niemandem ausreichend Informationen über die Erkrankung meiner Mutter/meines Vaters.	I don't get enough information from anyone about my mother's/father's illness.	general	1–101
STD_14	Ich weiß genau, an welche (professionellen) Stellen in mich wenden kann, wenn ich Hilfe wegen der Erkrankung meiner Mutter/meines Vaters benötige.	I know exactly which (professional) places I can turn to if I need help because of my mother's/father's illness.…	general	101–1
STD_15	Es gibt ausreichend Hilfsangebote für meine Eltern und mich.	There's enough help available for my parents and me.	general	101–1

*Items with scoring 1–101 are the normal coded items, with 1 meaning no agreement at all, 101 meaning full agreement. The higher the agreement the higher the stigma experiences. Items with scoring 101–1 are the reversed coded items, also referring to 101 as full agreement. The higher the agreement, the lower the stigma for these items*.

Table 3 shows our sample's scores on the final subscales and the overall COPMI-SQ instrument. Overall, the sample shows very similar average levels of family stigma on the subscales *Experienced SBA, Anticipated SBA, Affiliate Stigma* and the overall instrument. Here COPMI, with a mean score of 30, tend to be in the lower third of the possible values. On the *STD* subscale, COPMI are in the middle range of values. Supplementary Table 2 illustrates the intercorrelations of the final instrument scales. Overall, these tend to demonstrate small to very large correlations, all of which are significant.

**Table 3:** Sample characteristics revised instrument version COPMI-SQ.

**Scale**	***n***	**Min**	**Max**	**M**	**SD**
Experienced SBA	32	1.19	73.18	28.36	22.40
Anticipated SBA	32	1.00	73.94	34.59	25.28
Affiliate stigma	32	1.05	62.16	30.00	20.25
Structural discrimination	32	12.73	82.33	54.27	16.78
COPMI SQ total	32	7.64	66.24	35.50	18.36

*Scale characteristics of the sample on the revised subscales and the overall instrument COPMI-SQ after adjustments according to the item analyses*.

### Internal Reliability

Table 4 shows the internal consistencies for both the initial subscales and subscales adjusted by the item analyses as described above. The internal consistencies for the overall instrument are also visible in Table 4. We observed an improvement in all the scales' consistencies. We detected internal consistencies between α = 0.868 and α = 0.975 across the scales.

**Table 4:** Changes in the instrument structure through internal consistency analysis.

**Scale**	**α initial**	**α final**	**Initial number of items**	**Revised number of items**
Experienced SBA	0.940	0.948	18	17
Anticipated SBA	0.927	0.949	20	16
Affiliate stigma	0.904	0.933	23	19
Structural discrimination	0.817	0.868	31	15
COPMI-SQ—total	0.970	0.975	92	67

*Results of the consistency analysis. The initial internal consistency refers to all original items of a scale*.

## Discussion

This study reports on the development and piloting of a questionnaire to assess stigma in children of parents with a mental illness. The COPMI-SQ is the first instrument incorporating different stigma by association dimensions tailored to the stigma experiences of children of parents with a mental illness. After reducing the item number, the final version of the COPMI-SQ consists of 67 items representing four subscales: experienced stigma (17 items), anticipated stigma (16 items), self-stigma (19 items) and structural discrimination (15 items). The COPMI-SQ, once validated, can be used to better understand the complex phenomenon of stigma by association among young people and also to target the experiences and needs of this group in intervention studies or anti-stigma campaigns. As the questionnaire also captures which parental condition in which parent is present, it can be used to create insights to the extent to which stigma experiences vary for different parental disorders. In addition, it could potentially support shedding light on the gendered theoretical landscape of parenting.

### Developing the Instrument

The approach we took to develop the instrument followed scientifically accepted principles (20) by ensuring various construction phases. The construction process relied on (family) stigma theories (14, 20, 39, 60, 61), incorporated qualitative results from a comprehensive systematic review to best reflect the expressed stigma experiences of affected children (28), and included expert opinions on the initially constructed items. The COPMI-SQ is thus considered a solid basis for further reliability and validity studies.

### Item Reduction

We ran into difficulty with item analyses due to the fact that it was not a test construction for which clear rules could be applied while considering the required item difficulties or discriminatory power. Although recommendations could be used as a guide, each change or reduction of the item pool had to be made by carefully considering the content. For example, the subscale “Structural Discrimination” represents a very heterogeneous set of items, as they were meant to represent different institutional sources of discrimination, for example the educational system, as well as the media and the health care system. In this respect, we had to assume that discrimination perceived in one societal structure does not necessarily go hand in hand with a high level of discrimination in another, as there are unintended public and private sector policies that restrict opportunities for some groups (62) and we do not yet know precisely how they interact (62, 63). This means that the item-total correlations it did for not have to meet the same requirements as did the other subscales. We also had to make sure we did not over- or under-represent some sub-aspects.

Nevertheless, a detailed explanation of the reasons for deleting or retaining the items ensures transparency, and very high item-total correlations were achieved overall. Only in the “Structural Discrimination” subscale one item with a low item-total-correlation, namely “In the media, mental illness is appropriately portrayed” remained in the questionnaire. This can be justified due to the item's relevance to the content and that its discriminatory power of 0.282 was only minimally below the acceptable limit of 0.3. Nevertheless, items of low discriminatory power or showing low item-total correlations must be examined more closely in the further validation course in a factor analysis.

The very high values regarding internal reliability and the increase in Cronbach's alpha in each subscale, as well as in the final instrument compared to its first version suggest that we made the right decisions here.

We detected overall very strong correlations between the various subscales in the questionnaire's revised form. This may be an initial indication that also a one factor model may be considered. In a further validation study, factor structure and dimensionality have to be examined.

### Evaluating Reliability and Validity

The final COPMI-SQ's internal reliability can be considered as very good. It demonstrates good to excellent internal consistency in the subscales, as well as excellent internal reliability of the instrument as a whole. We ensured content validity through an expert panel, and evaluated the difficulty of understanding items with children themselves. However, due to the impossibility of conducting a factor analysis, whether these subscales are confirmed by the instrument's factor structure and multidimensionality cannot be assessed yet.

Test-retest reliability could not be assessed due to an insufficient sample size. However, we can assume that stigma experiences are also not constant variables. New experiences can always emerge, so that if test-retest reliability is to be measured, there should be a shorter time span between them. Furthermore, the mere questioning of these experiences could also lead to some young people, especially when confronted with the topic for the first time through the questionnaire, potentially being sensitized to subsequent experiences and accordingly revealing higher values at a later point in time.

### Strengths and Limitations

The greatest strength of the present study is that the COPMI-SQ was theory-based and developed supported by an extensive systematic review. In addition, the various steps in its development are presented transparently and in detail.

There are several areas for improvement and further investigations. About half of the participants reported having a mental illness of their own—a good representation of the population of children of parents with mental illness (64), but this means that their responses may be cofounded by experiencing stigma due to their own illness. Due to recruitment difficulties, we were unable to identify enough participants to permit a factor analysis and investigate multidimensionality and factor structure. The assumed four factors are not supported by the very high inter-correlations of the assumed subscales, but they cannot be ruled out either. However, the study is still ongoing. With the reduced number of items due to the item analyses, we can reach our sample size more easily. Other recruitment strategies, such as recruiting via schools, must be integrated in the future to reach this vulnerable target group. Since this paper is concerned with the methodology of developing a new instrument, content-related considerations of the different dimensions of stigma have been marginalized. A more detailed discussion of the meaning and interconnectedness of the different dimensions can be found in the paper reporting the results of the systematic review (28).

### Implications for Further Research and Use of the COPMI-SQ

In a further validation study, we will aim to recruit approximately 200 COPMI to examine our questionnaire's multidimensionality. In the first step, this should be examined via an exploratory factor analysis (EFA). An EFA is preferable to a confirmatory factor analysis (CFA), since it is possible that factor structures other than those expected might emerge (45). Especially as high inter-correlations between the subscales might indicate a one-factor structure, uni-dimensionality should also be checked. A CFA should then be conducted on a separate sample to confirm the structure of the scales resulting from the EFA.

A further validated COPMI-SQ could be used to help develop anti-stigma and general interventions for this population. Our first pilot data promises good reliability of the a priori assumed subscales. As our scale is constructed for young people aged 12–19, it can be used as a starting point to develop a scale addressing the same problem for younger children. A more creative and interactive way of getting those information from younger children, should be developed, to be able to incorporate younger children's views as well. Research in children's lived experiences is limited, and this is especially true for younger children.

## Data Availability Statement

The raw data supporting the conclusions of this article will be made available by the authors, without undue reservation.

## Ethics Statement

The studies involving human participants were reviewed and approved by Ethics Committee of the Psychology Department of Philipps University of Marburg. Written informed consent to participate in this study was provided by the participants' legal guardian/next of kin.

## Author Contributions

L-MD designed and completed the study, involved as the main person in charge in all development and analyses steps, and wrote the manuscript. MS co-organized the data collection process and supported the writing of the manuscript by reviewing and editing. KV assisted with data collection and data analysis. HC supervised the study and preparation of the manuscript and supported the writing of the manuscript by reviewing and editing.

## Funding

Funding was provided by the Working Group on Clinical Child and Adolescent Psychology, Department of Psychology, Philipps University of Marburg. The funding for the raffle was provided by COMPARE-family. COMPARE-family is funded by the Federal Ministry of Education and Research (BMBF) (01GL1748B). The funders did not influence the collection, analysis, and interpretation of data and played no role in writing the manuscript.

## Conflict of Interest

The authors declare that the research was conducted in the absence of any commercial or financial relationships that could be construed as a potential conflict of interest.

## Publisher's Note

All claims expressed in this article are solely those of the authors and do not necessarily represent those of their affiliated organizations, or those of the publisher, the editors and the reviewers. Any product that may be evaluated in this article, or claim that may be made by its manufacturer, is not guaranteed or endorsed by the publisher.
