# Supplementary material for: Children of Parents With a Mental Illness—Stigma Questionnaire: Development and Piloting
Source: Front Psychiatry. 2022 Apr 8;13:800037. doi: 10.3389/fpsyt.2022.800037 (PMC9023877; doi:10.3389/fpsyt.2022.800037)
Supplement: Supplementary file 1 [file Table_1.docx]

| **Supplementary Material**  **Table S1** Overview of the COPMI-SQ; item parameters; Item-total-correlations for initial and revised subscales, and reasons for retaining, rewording or deleting items | | | | | | | | | | | | | |
| --- | --- | --- | --- | --- | --- | --- | --- | --- | --- | --- | --- | --- | --- |
| **Subscale „Experienced SBA“** | |  | **Item parameters** | | | | | **Item-total-correlations**  **for initial subscale** |  | | | **Item-total-correlations for revised subscale** | |
| Original Item | Translation | Intended theoretical dimension | *n* | M | SD | $\sigma^{2}$ | *P_i_* | *r_it_* | *Consequence* | | *Explanation* | *r_it_* | |
| *Preceded by:*  ***Weil meine Mutter/mein Vater eine psychische Erkrankung hat, ….*** | *Preceded by:*  ***Because my mother/my father has a mental illness,…*** |  |  |  |  |  |  |  |  |  |  |  |  |
| ST05_01_ES  machen sich andere über meine Mutter/meinen Vater lustig. | *others make fun of my mother/father.* | hostile behaviors of others | 32 | 28.84 | 33.75 | 1139.36 | 27.84 | .702 | Retained | |  | .712 | |
| ST05_02_ES  reden andere hinter meinem Rücken über die Erkrankung meiner Mutter/meines Vaters. | *others talk about my mother's/father's illness behind my back.* | hostile behaviors of others | 32 | 34.44 | 36.19 | 1310.38 | 33.44 | .768 | Retained | |  | .767 | |
| ST05_03_ES  lästern andere über mich. | *others say awful things about me* | hostile behaviors of others | 32 | 25.63 | 30.68 | 941.66 | 24.63 | .853 | Retained | |  | .874 | |
| ST05_04_ES  lachen andere mich aus. | *others* ***laugh*** *at me.* | hostile behaviors of others | 32 | 22.87 | 30.93 | 957.08 | 21.88 | .731 | Retained | |  | .745 | |
| ST05_05_ES  tratschen andere das weiter. | *others gossip about it.* | hostile behaviours of others | 32 | 32.41 | 31.33 | 981.53 | 31.41 | .865 | Retained | |  | .878 | |
| ST05_06_ES  gehen andere mir aus dem Weg. | *others avoid me.* | Withdrawal and rejection | 32 | 30.06 | 34.64 | 1199.99 | 29.06 | .802 | Retained | |  | .808 | |
| ST05_07_ES  haben andere Angst vor meiner Mutter/meinem Vater oder mir. | *others are afraid of my mother/father or me.* | inappropriate language and contents | 32 | 24.69 | 27.50 | 756.48 | 23.69 | .787 | Retained | |  | .793 | |
| ST05_08_ES  möchten meine Freund*innen nicht mehr mit mir befreundet sein. | *my friends no longer want to be friends with me.* | Withdrawal and rejection | 32 | 18.03 | 24.81 | 615.52 | 17.03 | .706 | Retained | | Low difficulty. but high enough inter-item-correlations. relevant in terms of content | .706 | |
| ST05_09_ES  wollen sich meine Mitschüler*innen / Kommiliton*innen/ Arbeitskolleg*innen nicht mehr mit mir treffen. | *my classmates colleagues/ work colleagues no longer want to get together with me.* | Withdrawal and rejection | 32 | 19.63 | 25.52 | 651.15 | 18.63 | .641 | Retained | |  | .644 | |
| ST05_10_ES  ärgern mich meine Mitschüler*innen/ Kommiliton*innen/ Arbeitskolleg*innen. | *my classmates/colleagues/work colleagues aggravate me.* | hostile behaviors of others | 32 | 24.41 | 29.48 | 869.60 | 23.41 | .883 | Retained | |  | .905 | |
| ST05_11_ES  werde ich in der Schule / Uni / auf der Arbeit gemobbt. | *I‘m bullied at school/university/work.* | hostile behaviors of others | 32 | 18.28 | 24.94 | 621.95 | 17.28 | .783 | Retained | | Low difficulty. but high enough inter-item-correlations; relevant in terms of content | .806 | |
| ST05_12_ES  wussten andere nicht, wie sie passend darauf reagieren / damit umgehen sollten. | *others did not know how to react to or deal with it appropriately.* | inappropriate language and contents | 32 | 44.19 | 32.13 | 1032.41 | 43.19 | .574 | Retained | |  | .556 | |
| ST05_13_ES  haben andere mir geraten, selbst keine Kinder zu bekommen. | *others have advised me not to have children myself.* | inappropriate language and contents | 32 | 16.94 | 24.80 | 614.77 | 15.94 | .688 | Retained | |  | .707 | |
| ST05_14_ES  haben andere verletzende Sachen über mich oder meine Mutter/meinen Vater gesagt. | *others have said hurtful things about me or my mother/father.* | Hostile behaviours of others | 32 | 30.94 | 33.09 | 1095.28 | 29.94 | .836 | Retained | |  | .834 | |
| ST06_13_Invert_ES  INVERTIERT es gibt Leute, mit denen ich über meine Ängste und Sorgen reden kann. | *There are people I can talk to about my fears and worries.* | Withdrawal and rejection | 32 | 34.91 | 31.43 | 988.08 | 33.91 | .408 | Retained | |  | .395 | |
| ***Separate items without an item root*** | |  |  |  |  |  |  |  |  |  |  |  | |
| ST06_14_ES  Andere Leute möchten nicht mit mir über die Erkrankung meiner Mutter/meines Vaters sprechen. | *Other people don't want to talk to me about my mother's/father's illness.* | withdrawal and rejection | 32 | 40.53 | 29.31 | 859.41 | 39.53 | .481 | Retained | |  | .466 | |
| ST06_15_ES  Ich kann mit anderen Verwandten offen über die Erkrankung meiner Mutter/meines Vaters sprechen. | *I can talk openly with other relatives about my mother's/father's illness.* | Withdrawal and rejection | 32 | 54.44 | 32.87 | 1080.38 | 53.44 | .136 | **Deleted**  after phase 1  (1) | | Lowest inter-item-correlation.content-wise deleted | - |  |
| ST06_16_Invert_ES  INVERTIERT: Wenn ich wegen der Erkrankung meiner Mutter/meines Vaters Hilfe brauche, gibt es Personen. mit denen ich sprechen kann. | *If I need help because of my mother's/father's illness. there are people I can turn to.* | Withdrawal and rejection | 32 | 35.31 | 31.56 | 996.02 | 34.31 | .448 | Retained | |  | .415 | |
|  | | | | | | | | | | | | | |
| **Subscale „Anticipated Stigma“** | |  |  |  |  |  |  |  |  |  |  |  | |
| Preceded by:  **Wenn andere von der Erkrankung meiner Mutter/meines Vaters erfahren würden**,… | *Preceded by:*  ***If others found out about my mother's/ father's illness,…*** |  |  |  |  |  |  |  |  |  |  |  | |
| ST03_01_AS  würden sie sich über meine Mutter/meinen Vater lustig machen. | *they’d make fun of my mother/father.* | Fearing hostile behaviors | 32 | 38.88 | 35.52 | 1262.04 | 37.88 | .765 | Retained | |  | .792 | |
| ST03_02_AS  würden sie hinter meinem Rücken schlecht über die Erkrankung meiner Mutter/meines Vaters reden. | *they’d talk badly about my mother's/father's illness behind my back* | Fearing hostile behaviors | 32 | 44.16 | 37.98 | 1443.10 | 43.16 | .809 | Retained | |  | .807 | |
| ST03_03_AS  würden sie über mich lästern. | *they‘d bad-mouth me.* | Fearing hostile behaviors | 32 | 41.53 | 36.46 | 1329. 48 | 40.53 | .844 | Retained | |  | .877 | |
| ST03_04_Invert_AS  INVERTIERT würde das an ihrem Verhalten mir gegenüber nichts ändern | *it would n’t change their behavior towards me* | Fearing hostile behaviors | 32 | 45.59 | 35.42 | 1254.57 | 44.59 | .410 | Retained | |  | .426 | |
| ST03_05_AS  würden sie mich auslachen. | *they’d laugh at me.* | Fearing hostile behaviors | 32 | 29.59 | 35.45 | 1257.08 | 28.59 | .765 | Retained | |  | .781 | |
| ST03_06_Invert_AS  INVERTIERT würden sie das für sich behalten. | *they would keep it to themselves.* | Fearing hostile behaviors | 32 | 42.28 | 31.95 | 1020.85 | 41.28 | .501 | Retained | |  | .484 | |
| ST03_07_AS  würden sie mir aus dem Weg gehen. | *they‘d avoid me* | Fearing lack of understanding& rejection | 32 | 32.38 | 32.41 | 1050.88 | 31.38 | .812 | Retained | |  | .852 | |
| ST03_08_AS  würden sie Angst vor meiner Mutter/meinem Vater oder mir bekommen | *they‘d become afraid of my mother/father or me* | Fearing of negative attitudes and ascriptions | 32 | 40.91 | 35.44 | 1256.15 | 39.91 | .565 | Retained | |  | .575 | |
| ST03_09_AS  INVERTIERT würden meine Freund*innen weiterhin mit mir befreundet sein wollen. | *my friends would still want to be friends with me.* | Fearing lack of understanding& rejection | 32 | 36.12 | 36.72 | 1348.63 | 35.12 | .161 | **Deleted**  after Phase 1  **(3)** | | Low inter-item-correlation; content-wise not needed | - | |
| ST03_10_AS  würden sich meine Mitschüler*innen/ Kommiliton*innen/ Arbeitskolleg*innen nicht mit mir... | *my classmates/ fellow students/ colleagues would no longer want to get together with me.* | Fearing lack of understanding& rejection | 32 | 27.81 | 32.56 | 1060.28 | 26.81 | .545 | Retained | |  | .585 | |
| ST03_11_AS  würden mich meine Mitschüler*innen/ Kommiliton*innen/ Arbeitskolleg*innen ärgern. | *my classmates/fellow students/ colleagues at work would get angry with me.* | Fearing hostile behaviors | 32 | 27.19 | 28.94 | 837.57 | 26.19 | .866 | Retained | |  | .872 | |
| ST03_12_AS  würde ich in der Schule / Uni / auf der Arbeit gemobbt werden. | *I’d be bullied at school/university/work.* | Fearing hostile behaviors | 32 | 26.75 | 28.45 | 809.61 | 25.75 | .879 | Retained | |  | .866 | |
| ST03_13_AS  INVERTIERT wüssten sie, wie sie passend darauf reagieren/ damit umgehen sollten. | *they‘d know how to react to or handle it appropriately.* | Fearing lack of understanding& rejection | 32 | 63.38 | 29.21 | 853.21 | 62.38 | .129 | **Deleted**  after Phase 1  **(2)** | | Lowest inter-item-correlation | - | |
| ST03_14_AS  würden andere mir raten, selbst keine Kinder zu bekommen. | *others would advise me not to have children myself.* | Fearing of negative attitudes and ascriptions | 32 | 26.34 | 30.16 | 910.03 | 25.34 | .826 | Retained | |  | .820 | |
| ST03_15_AS  würden sie verletzende Sachen über mich oder meine Mutter/ meinen Vater sagen. | *they‘d say hurtful things about me or my mother/father.* | Fearing hostile behaviors | 32 | 37.56 | 35.57 | 1265.28 | 36.56 | .879 | Retained | |  | .866 | |
| Preceded by:  **Wenn Fachleute (Jugendamt/Psycholog*innen/Sozialarbeiter*innen. etc.) von der Erkrankung meiner Mutter/meines Vaters erfahren würden. …** | *Preceded by:*  ***If professionals (youth welfare office/psychologists/social workers. etc.) found out about my mother's/father's illness.*** *...* |  |  |  |  |  |  |  |  |  |  |  | |
| ST04_01_Invert_AS  INVERTIERT könnte ich weiterhin zu Hause wohnen bleiben. | *I could still keep living at home.* | Fearing of negative attitudes and ascriptions | 32 | 34.66 | 37.65 | 1418.03 | 33.66 | .622 | Retained | |  | .606 | |
| ST04_02_AS  würde ich von meiner Mutter/meinem Vater getrennt werden. | *I’d be separated from my mother/father.* | Fearing of negative attitudes and ascriptions | 32 | 15.72 | 23.75 | 563.89 | 14.72 | .267 | **Deleted**  after Phase 1  **(1)** | | conspicuous both in difficulty and low discriminatory power | - | |
| ST04_03_AS  würde ich die Situation zu Hause verschweigen oder verharmlosen aus Angst, von meiner Mutter/ m... | *I’d keep quiet or downplay the situation at home for fear of being separated from my mother/father.* | Fearing of negative attitudes and ascriptions | 32 | 35.63 | 37.47 | 1404.11 | 34.63 | .172 | **Deleted**  After Phase 1  **(4)** | | Low inter-item-correlation; sentence too long and complicated | - | |
| ST05_26_AS  bringe ich selten neue Freund*innen mit nach Hause, aus Angst. dass sie dann nicht mehr mit mir befreundet sein wollen. | I rarely bring new friends home for fear that they won't want to be friends with me anymore. | Fearing lack of understanding and rejection | 32 | 22.22 | 25.51 | 651.20 | 21.22 | .633 | Retained | |  | .602 | |
| **Seperate item without an item root** | |  |  |  |  |  |  |  |  |  |  |  | |
| ST06_17_Invert_AS  INVERTIERT Ich habe kein Problem damit, meinen Freund*innen meine (erkrankte) Mutter/meinen (erkrankten) Vater vorzustellen. | *I have no problem introducing my ( ill) mother/father to my friends.* | Fearing any harmful reaction | 32 | 35.53 | 34.87 | 1216.06 | 34.53 | .674 | Retained | |  | .707 | |
|  | | | | | | | | | | | | | |
| **Subscale „Affiliate stigma“** | |  |  |  |  |  |  |  |  |  |  |  | |
| Preceded by:  **Weil meine Mutter/mein Vater eine psychische Erkrankung hat, …** | *Preceded by:*  ***Because my mother / my father has a mental illness, …*** |  |  |  |  |  |  |  |  |  |  |  | |
| ST05_15_SeS  denke ich. mit mir stimmt etwas nicht. | *I think there’s something wrong with me.* | Beliefs of being inferior | 32 | 38.88 | 32.02 | 1025.27 | 37.88 | .787 | Retained | |  | .820 | |
| ST05_16_SeS  versuche ich. mich besonders normal und unauffällig zu verhalten, damit andere mir nicht anmerken. dass ich nicht normal bin. | *I try to act particularly normal and inconspicuous so that others don’t notice that I’m not normal.* | Beliefs of being contaminated | 32 | 41.78 | 36.31 | 1318.56 | 40.78 | .680 | Retained | |  | .717 | |
| ST05_17_SeS  nehmen andere wahr, dass ich anders / komisch bin. | *others notice that I‘m different/weird.* | Beliefs of being contaminated | 32 | 29.78 | 33.40 | 1115.85 | 28.78 | .447 | Retained | |  | .491 | |
| ST05_18_SeS  versuche ich so viel Zeit wie möglich außer Haus/mit anderen Leuten zu verbringen, damit ich eine normale/gesunde Person werden kann. | *I try to spend as much time as possible out of the house/with other people so that I can become a normal/healthy person.* | Beliefs of being contaminated | 32 | 32.75 | 29.95 | 896.84 | 31.75 | .361 | **Deleted**  after Phase 2 | | Low inter-item-correlation for total instrument; complicated wording | - | |
| ST05_19_SeS  habe ich Angst, mich anstecken zu können. | *I’m afraid of catching the illness.* | Beliefs of being contaminated | 32 | 21.06 | 22.46 | 504.51 | 20.06 | .478 | Retained | |  | .505 | |
| ST05_20_SeS  denke ich bei kleinsten Anzeichen, dass ich dieselbe Erkrankung habe wie meine... | *I think at the slightest sign that I have the same illness as my mother/father.* | Beliefs of being contaminated | 32 | 31.5 | 32.29 | 1042.90 | 30.5 | .795 | Retained | |  | .783 | |
| ST05_21_SeS  denke ich, dass ich später keine eigenen Kinder bekommen sollte. | *I don’t think I should have children of my own later on.* | Beliefs of being contaminated | 32 | 16 | 20.40 | 416.32 | 15 | .583 | Retained | | Low difficulty. but seems important to capture „contamination” beliefs | .575 | |
| ST05_22_SeS  fühle ich mich weniger wert. | *I feel less worthy.* | Beliefs of being inferior | 32 | 24.91 | 28.87 | 833.63 | 23.91 | .655 | Retained | |  | .676 | |
| ST05_23_SeS  ist meine Familie nicht richtig. | *my family is not right.* | Beliefs of being inferior | 32 | 27.41 | 26.26 | 689.60 | 26.41 | .528 | Retained | |  | .550 | |
| ST05_24_SeS  fühle ich mich schuldig. | *I feel guilty.* | Beliefs of being inferior | 32 | 37.72 | 34. 59 | 1196.46 | 36.72 | .666 | Retained | |  | .687 | |
| ST05_25_SeS  schäme ich mich. | *I feel ashamed.* | Beliefs of being inferior | 32 | 31.34 | 27.25 | 742.68 | 30.34 | .612 | Retained | |  | .633 | |
| **Seperate items without an item root** | |  |  |  |  |  |  |  |  |  |  |  | |
| ST06_01_Invert_SeS  INVERTIERT Die Erkrankung meiner Mutter/meines Vaters hat keinen Einfluss auf meine Pläne, später eigene Kinder zu haben. | *My mother's/father's illness has no influence on my plans to have children of my own later.* | Beliefs of being contaminated | 32 | 47.16 | 40.93 | 1675.36 | 46.16 | -.112 | **Deleted**  after Phase 1  (1) | | Negative inter-item-correlation; wording potentially misleading – is also captured by 05_21 | - | |
| ST06_02_SeS  Ich fühle mich als würde ich ein Schild mit mir herumtragen: „Er/Sie hat eine Mutter/einen Vater mit einer psychischen Erkrankung“ | *I feel like I'm carrying around a sign: "He/she has a mother/father with a mental illness".* | Beliefs of being contaminated | 32 | 17.03 | 23.37 | 546.29 | 16.03 | .497 | Retained | | Low difficulty. but adopted from a validated scale; seems important to capture “contamination” beliefs | .515 | |
| ST06_03_Invert_SeS  INVERTIERT Ich bin ein ganz normales Kind wie jedes andere auch. | *I’m just a normal kid like any other.* | Beliefs of being inferior | 32 | 31 | 26.70 | 712.96 | 30 | .499 | Retained | |  | .449 | |
| ST06_04_SeS  Weil ich so ein schwieriges Kind bin, ist meine Mutter/ ist mein Vater erkrankt. | *Because I’m such a difficult child. my mother/ father has become ill.* | Beliefs of being inferior | 32 | 22.09 | 27.81 | 773.50 | 21.09 | .539 | Retained | |  | .524 | |
| ST06_05_SeS  INVERTIERT Ich kann nichts für die Erkrankung meiner Mutter/meines Vaters. | *It is not my fault that my mother/father is ill.* | Beliefs of being inferior | 32 | 43.06 | 39.95 | 1596.13 | 42.06 | .149 | **Deleted**  after Phase 1  (2) | | Low inter-item-correlation | - | |
| ST06_06_SeS  Ich bin (mit-)verantwortlich dafür, dass sich der Zustand meiner Mutter/meines Vaters nicht verbessert. | *I’m (co-)responsible for the fact that the condition of my mother/father isn’t improving.* | Beliefs of being inferior | 32 | 29.28 | 29.66 | 879.69 | 28.28 | .682 | Retained | |  | .677 | |
| ST06_07_SeS  INVERTIERT Ich rede offen mit Freund*innen über die Erkrankung meiner Mutter/meines Vaters. | *I talk openly with friends about my mother's /father's illness.* | Beliefs of being inferior | 32 | 65.69 | 32.38 | 1048.61 | 64.69 | .133 | **Deleted**  after Phase 1  (3) | | Low inter-item-correlation; likely that it measures sth else | - | |
| ST06_08_SeS  Ich muss die Erkrankung meiner Mutter/meines Vaters geheim halten. | *I have to keep my mother's/father's illness a secret.* | Beliefs of being inferior | 32 | 30.06 | 31.75 | 1008.44 | 29.06 | .586 | Retained | |  | .597 | |
| ST06_09_SeS  Wenn ich die Erkrankung meiner Mutter/meines Vaters beschreibe, spiele ich die Schwere der Erkrankung heru... | *When I describe my mother's/father's illness. I downplay its severity* | Beliefs of being inferior | 32 | 34.72 | 32.16 | 1034.53 | 33.72 | .709 | Retained | |  | .716 | |
| ST06_10_SeS  Mir ist es peinlich, dass meine Mutter/mein Vater eine psychische Erkrankung hat. | *I’m embarrassed that my mother/father has a mental illness.* | Beliefs of being inferior | 32 | 28.13 | 33.43 | 1117.79 | 27.13 | .680 | Retained | |  | .667 | |
| ST06_11_SeS  Ich schäme mich dafür, dass meine Mutter/mein Vater nicht wie andere Mütter/Väter ist. | *I’m ashamed that my mother/father isn’t like other mothers/fathers* | Beliefs of being inferior | 32 | 29.63 | 31.08 | 966.50 | 28.63 | .712 | Retained | |  | .705 | |
| ST06_12_SeS  Wenn meine Mutter/mein Vater wegen ihrer/seiner Erkrankung verurteilt wird, fühle ich mich auch verurteilt. | *If my mother/father is judged because of her/his illness. I feel judged too.* | Beliefs of being contaminated | 32 | 47.88 | 34.95 | 1221.46 | 46.88 | .619 | Retained | |  | .655 | |
|  | | | | | | | | | | | | | |
| **Subscale „Structural Discrimination“** | |  |  |  |  |  |  |  |  |  |  |  | |
| Preceded by (after an filter question whether the parent ever has been hospitalized before):  **Wenn meine Mutter/ mein Vater aufgrund der psychischen Erkrankung im**  **Krankenhaus war, …** | *Preceded by:*  ***When my mother/father was in hospital because of the mental illness,…*** |  |  |  |  |  |  |  |  |  |  |  | |
| ST08_01_Invert_STD  INVERTIERT konnte ich das Personal immer ansprechen, wenn ich Fragen zur Erkrankung meiner Mutter/meines Vaters hatte. | *I could always approach the staff if I had any questions about my mother's/father's illness.* | Health care system | 18 | 53.83 | 44.04 | 1939.79 | 52.83 | .424 | Retained | |  | .583 | |
| ST08_02_STD  hätte ich gerne mehr Informationen vom Krankenhauspersonal bekommen. | *I’d have liked to get more information from the hospital staff.* | Health care system | 18 | 36.17 | 36.81 | 1355.21 | 35.17 | .474 | Retained | |  | .594 | |
| ST08_03_Invert_STD  INVERTIERT fühlte ich mich vom Krankenhauspersonal gut einbezogen und informiert. | *I felt well integrated and informed by the hospital staff.* | Health care system | 18 | 54.44 | 37.34 | 1394.03 | 53.44 | .388 | Retained | |  | .564 | |
| ST08_04_STD  empfand ich die Atmosphäre/Umgebung als kalt. | *I found the atmosphere/environment cold.* | Health care system | 18 | 39.78 | 38.63 | 1492.42 | 38.78 | .044 | **Deleted**  after Phase 1  (4) | | inter-item-correlation close to 0; probably measures sth else; was already discussed when generating items | - | |
| ST08_05_STD  fühlte ich mich wegen der Einrichtung im Krankenhaus dort unwohl. | *I felt uncomfortable there because of the facilities in the hospital.* | Health care system | 18 | 36.22 | 33.46 | 1119.83 | 35.22 | .186 | **Deleted**  After Phase 1  (5) | | inter-item-correlation close to 0; probably measures sth else; was already discussed when generating items | - | |
| ST08_06_STD  fühlte ich mich, als wäre ich dort unerwünscht. | *I felt like I was unwanted there.* | Health care system | 18 | 36.44 | 36.91 | 1362.61 | 35.44 | .494 | Retained | |  | .392 | |
| ST08_07_Invert_STD  INVERTIERT schätzte das Krankenhauspersonal mein Wissen über die Erkrankung meiner Mutter/meines Vaters. | *the hospital staff appreciated my knowledge about my mother's/father's illness.* | Health care system | 18 | 58.06 | 36.16 | 1307.59 | 57.06 | .201 | **Deleted**  after Phase 1  (10) | | Low inter-item-correlation; mental health system still over-represented in STD | - | |
| ST08_08_Invert_STD  INVERTIERT war die Beziehung zwischen mir und dem Krankenhauspersonal gut. | *the relationship between me and the hospital staff was good.* | Health care system | 18 | 55.94 | 34.57 | 1194.76 | 54.94 | .528 | Retained | |  | .617 | |
| ST08_09_Invert_STD  INVERTIERT war das Krankenhauspersonal nett zu mir. | *the hospital staff were kind to me.* | Health care system | 18 | 42.72 | 35.10 | 1232.10 | 41.72 | .279 | **Deleted**  after Phase 2 | | Even lower inter-item-correlation when analysing total instrument | - | |
| ST08_10_STD  verhielt sich das Krankenhauspersonal mir gegenüber unsensibel. | *the hospital staff behaved insensitively towards me.* | Health care system | 18 | 25 | 27.99 | 783.41 | 24 | .320 | **Deleted**  after Phase 1  (14) | | Relatively low difficulty. mental health system still over-represented in STD | - | |
| ST08_11_STD  verhielt sich das Krankenhauspersonal meiner Mutter/meinem Vater gegenüber unsensibel. | *the hospital staff behaved insensitively towards my mother/father.* | Health care system | 18 | 18.89 | 21.29 | 453.28 | 17.89 | .299 | **Deleted**  After phase 1  (7) | | Low difficulty and inter-item-correlation; mental health system still over-represented in STD | - | |
| ST08_12_Invert_STD  INVERTIERT ging es ihr/ihm danach besser. | *she/he felt better afterwards.* | Health care system | 18 | 34.11 | 28.46 | 810.22 | 33.11 | -.028 | **Deleted**  after Phase 1  (3) | | Negative inter-item-correlation; probably measures sth else | - | |
| ST08_13_Invert_STD  INVERTIERT wurde sie/er erst entlassen. wenn es ihr/ihm besser ging. | *she/he was only discharged when she/he was better.* | Health care system | 18 | 33.11 | 26.01 | 676.34 | 32.11 | -.144 | **Deleted**  after Phase 1  (1) | | Negative inter-item-correlation; probably measures sth else | -- | |
| **Seperate items without an item root** | |  |  |  |  |  |  |  |  |  |  |  | |
| ST09_01_STD  Meiner Mutter/meinem Vater wurde durch das Gesundheitssystem nicht genug geholfen. | *My mother/father wasn’t helped enough by the health system.* | Health care system | 18 | 46.75 | 32.17 | 1034.71 | 45.75 | .451 | Retained | |  | .438 | |
| ST09_02_Invert_STD  INVERTIERT Meine Mutter/mein Vater erhält eine medizinische/psychologische Behandlung in dem Ausmaß, in dem sie/er sie braucht. | *My mother/father receives medical/psychological treatment to the extent she/he needs it.* | Health care system | 18 | 50.16 | 32.21 | 1037.30 | 49.16 | .204 | **Deleted**  after Phase 1  (12) | | Low inter-item-correlation; rather concerns the experiences of the affected parent in terms of content | - | |
| ST09_03_STD  Ich bin oft frustriert, weil meine Mutter/ mein Vater nicht angemessen medizinisch... | *I’m often frustrated because my mother/father doesn’ get adequate medical...* | Health care system | 18 | 33.97 | 31.02 | 961.98 | 32.97 | .227 | **Deleted**  after Phase 1  (13) | | Low inter-item-correlation; rather concerns the experiences of the affected parent in terms of content | - | |
| Preceded by:  **In der Schule…** | *Preceded by:*  ***At school.…*** |  |  |  |  |  |  |  |  |  |  |  | |
| ST10_01_Invert_STD  INVERTIERT wird man gut über psychische Erkrankungen informiert. | *you’re well informed about mental illness.* | Health care system | 18 | 81.25 | 27.95 | 781.36 | 80.25 | .229 | **Deleted**  after Phase 1  (11) | | High difficulty. low inter-item-correlation | - | |
| ST10_02_STD  würde ich gerne mehr über psychische Erkrankungen erfahren. | *I’d like to learn more about mental illness.* | Educational system | 18 | 61.84 | 35.92 | 1290.52 | 60.84 | .295 | Retained | | Low inter-item-correlation. but on the edge of acceptable corr.. seems important in terms of possible interventions | .395 | |
| ST10_03_Invert_STD  INVERTIERT fragen mich meine Lehrer*innen manchmal, wie es meiner Mutter/meinem Vater geht. | *my teachers sometimes ask me how my mother/father is doing.* | Educational system | 18 | 84.16 | 28.72 | 824.98 | 83.16 | -.124 | **Deleted**  after Phase 1  (2) | | Low inter-item-correlation; high difficulty; probably measures sth else | - | |
| ST10_04_Invert_STD  INVERTIERT kann ich mit meinen Lehrer*innen über die Erkrankung meiner Mutter/meines Vaters sprechen. | *I can talk to my teachers about my mother's or father's illness.* | Educational system | 18 | 67.63 | 35.12 | 1233.53 | 66.63 | .680 | Retained | |  | .548 | |
| ST10_05_Invert_STD  INVERTIERT gehen die Lehrer*innen auf mich und meine Schwierigkeiten zu Hause ein. | *the teachers respond to me and my difficulties at home.* | Educational system | 18 | 78.69 | 25.56 | 653.32 | 77.69 | .434 | Retained | |  | .361 | |
| ST10_06_Invert_STD  INVERTIERT fühle ich mich wegen der Erkrankung meiner Mutter/meines Vaters nicht benachteiligt. | *I don’t feel disadvantaged because of my mother's/father's illness.* | Educational system | 18 | 29.31 | 31.52 | 993.64 | 28.31 | .601 | Retained | |  | .705 | |
| Preceded by:  **In den Medien…** | *Preceded by:*  ***In the media,*** |  |  |  |  |  |  |  |  |  |  |  | |
| ST11_01_STD  habe ich schon einmal etwas über Personen, die psychisch erkrankt sind, gesehen oder gelesen, das ich verletzend fand. | *I‘ve already seen or read something about mentally ill people that I found hurtful.* | Media | 18 | 62.81 | 35.20 | 1239.13 | 61.81 | .258 | **Deleted**  after Phase 1  (9) | | Low inter-item-correlation | - | |
| ST11_02_Invert_STD  INVERTIERT werden psychische Erkrankungen angemessen dargestellt. | *Mental illness is portrayed appropriately.* | Media | 18 | 70.09 | 20.07 | 402.80 | 69.09 | .392 | Retained | | Low inter-item-correlation for final scale; but keeping item ensuring media is still represented | .282 | |
| ST11_03_STD  sollte das Thema psychischer Erkrankungen sensibler dargestellt werden. | *The topic of mental illness should be presented more sensitively.* | Media | 18 | 65.47 | 24.14 | 582.84 | 64.47 | .107 | **Deleted**  after Phase 1  (6) | | inter-item-correlation close to 0 | - | |
| ST11_04_STD  werden falsche Informationen über psychische Erkrankungen vermittelt. | *False information about mental illness is conveyed.* | Media | 18 | 57.47 | 22.58 | 509.81 | 56.47 | .220 | **Deleted**  after Phase 1  (15) | | Low inter-item-correlation | - | |
| ST11_05_STD  werden psychische Erkrankungen negativ dargestellt, was dazu beiträgt, dass viele Leute Vorurteile entwickeln. | *Mental illness is portrayed negatively, which contributes to many people developing prejudices.* | Media | 18 | 70.84 | 24.49 | 599.75 | 69.84 | .199 | **Reworded:**  *“Mental illness is portrayed negatively.”* | | To avoid multidimensionality within one item | .319 | |
| **Seperate items without an item root** | |  |  |  |  |  |  |  |  |  |  |  | |
| ST12_01_STD  Ich erhalte von niemandem ausreichend Informationen über die Erkrankung meiner Mutter/meines Vaters. | *I don’t get enough information from anyone about my mother's/father's illness.* | General | 18 | 30.06 | 26.77 | 716.45 | 29.06 | .702 | Retained | |  | .635 | |
| ST12_02_STD  Niemand scheint über psychische Erkrankungen zu sprechen. | *Nobody seems to talk about mental illness.* | General | 18 | 49.25 | 31.01 | 961.42 | 48.25 | .258 | **Deleted**  after Phase 1  (8) | | Low inter-item-correlation; was already discussed in phase of item generation because of expected vagueness | - | |
| ST12_03_Invert_STD  Ich weiß genau, an welche (professionellen) Stellen in mich wenden kann, wenn ich Hilfe wegen der Erkrankung meiner Mutter/meines Vaters benötige. | *I know exactly which (professional) places I can turn to if I need help because of my mother's/father's illness.…* | General | 18 | 50.56 | 35.25 | 1242.19 | 49.56 | .554 | Retained | |  | .554 | |
| ST12_04_Invert_STD  Es gibt ausreichend Hilfsangebote für meine Eltern und mich. | *There’s enough help available for my parents and me.* | General | 18 | 44.41 | 27.38 | 749.80 | 43.41 | .747 | Retained | |  | .689 | |
