# Supplementary material for: Children of Parents With a Mental Illness—Stigma Questionnaire: Development and Piloting
Source: Front Psychiatry. 2022 Apr 8;13:800037. doi: 10.3389/fpsyt.2022.800037 (PMC9023877; doi:10.3389/fpsyt.2022.800037)
Supplement: Supplementary file 2 [file Table_2.docx]

| **Table S2.** *Intercorrelations between COPMI-SQ- (sub)scales* | | | | | |
| --- | --- | --- | --- | --- | --- |
| Scale | 1 | 2 | 3 | 4 | 5 |
| 1. Experienced SBA | -- | .776^**^ | .811^**^ | .477^**^ | .938^**^ |
| 1. Anticipated SBA | .776^**^ | -- | .658^**^ | .398^*^ | .855^**^ |
| 1. Affiliate Stigma | .811^**^ | .658^**^ | -- | .552^**^ | .901^**^ |
| 1. Structural discrimination | .477^**^ | .398^*^ | .552^**^ | -- | .630^**^ |
| 1. COPMI-SQ | .938^**^ | .855^**^ | .901^**^ | .630^**^ | -- |

| *Note*. Correlations according to Spearman (*r_s_*) at T1, *n* = 32.  ** The correlation is significant at the 0.01 level (two-sided).  * The correlation is significant at the 0.05 level (two-sided). |
| --- |
